# The high-affinity tryptophan uptake transport system in human cells

**DOI:** 10.1042/BST20230742

**Published:** 2024-05-30

**Authors:** Keisuke Wakasugi, Takumi Yokosawa

**Affiliations:** 1 Komaba Organization for Educational Excellence, The University of Tokyo, 3-8-1 Komaba, Meguro-ku, Tokyo 153-8902, Japan; 2 Department of Life Sciences, Graduate School of Arts and Sciences, The University of Tokyo, 3-8-1 Komaba, Meguro-ku, Tokyo 153-8902, Japan; 3 Department of Biological Sciences, Graduate School of Science, The University of Tokyo, 7-3-1 Hongo, Bunkyo-ku, Tokyo 113-0033, Japan

**Keywords:** aminoacyl-tRNA synthetase, indoleamine 2, 3-dioxygenase, interferon-γ, tryptophan, tryptophanyl-tRNA synthetase

## Abstract

The L-tryptophan (Trp) transport system is highly selective for Trp with affinity in the nanomolar range. This transport system is augmented in human interferon (IFN)-γ-treated and indoleamine 2,3-dioxygenase 1 (IDO1)-expressing cells. Up-regulated cellular uptake of Trp causes a reduction in extracellular Trp and initiates immune suppression. Recent studies demonstrate that both IDO1 and tryptophanyl-tRNA synthetase (TrpRS), whose expression levels are up-regulated by IFN-γ, play a pivotal role in high-affinity Trp uptake into human cells. Furthermore, overexpression of tryptophan 2,3-dioxygenase (TDO2) elicits a similar effect as IDO1 on TrpRS-mediated high-affinity Trp uptake. In this review, we summarize recent findings regarding this Trp uptake system and put forward a possible molecular mechanism based on Trp deficiency induced by IDO1 or TDO2 and tryptophanyl-AMP production by TrpRS.

## Introduction

**Figure 1.**
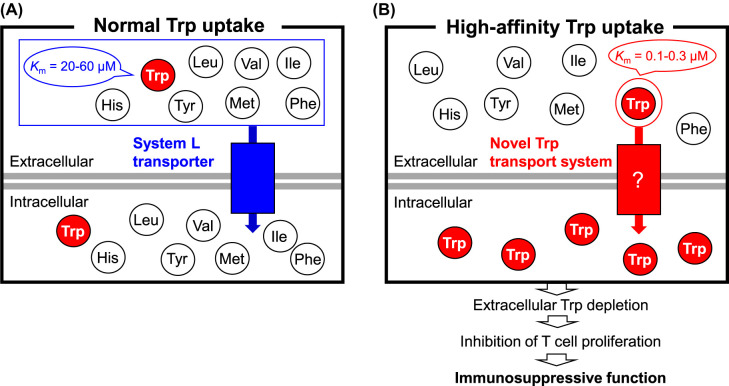
Comparison between normal Trp uptake mediated by System L transporter and high-affinity Trp uptake into cells. (**A**) Normal Trp uptake mediated by System L transporter. (**B**) High-affinity Trp uptake mediated by a novel Trp transporter system.

Mammalian cells cannot synthesize L-tryptophan (Trp). Given that Trp does not freely diffuse across the cell membrane it must be transported. Uptake of large hydrophobic amino acids with branched/aromatic side chains into mammalian cells is mediated by the ubiquitous System L transporter [1–3]. Thus, the System L transporter is responsible for the uptake of not only Trp but also L-histidine (His), L-isoleucine (Ile), L-leucine (Leu), L-methionine (Met), L-phenylalanine (Phe), Trp, L-tyrosine (Tyr), and L-valine (Val) (Figure 1A). As such, this transport system cannot selectively uptake Trp. System L is heterodimeric and comprises a surface antigen 4F2 heavy chain along with a catalytic light chain i.e. either L-type amino acid transporter 1 (LAT1) or 2 (LAT2) [1–3]. The affinity of these transporters for Trp, expressed as the Michaelis–Menten constant *K_m_*, is 20–60 μM [1–3] and the plasma concentration of Trp is typically ∼50 μM (Figure 1A) [4]. System L operates independently of the transmembrane Na^+^ gradient for the transportation of amino acids and its function can be selectively inhibited with 2-aminobicyclo-(2,2,1)-heptane-2-carboxylic acid (BCH) [1–3].

More recently, a distinct amino acid transport system with high affinity and selectivity for Trp was identified in interferon (IFN)-γ-treated and indoleamine 2,3-dioxygenase 1 (IDO1)-expressing cells [5–8]. Trp uptake into cells such as monocyte-derived macrophages (MDMs) and those with high IDO1 expression levels is characterized by a high degree of specificity and affinity for Trp (*K_m_* for Trp is 0.1–0.3 μM), with no observed uptake of other neutral amino acids (e.g. Tyr or Phe) (Figure 1B) [5,8]. Furthermore, this novel Trp uptake mechanism, which does not require a transmembrane Na^+^ gradient, is not inhibited by other amino acids or by BCH [5]. These observations indicate that the transporter operates via a mechanism independent of system L.

Here, we review new insights regarding the underlying mechanism of high-affinity Trp uptake. In particular, we introduce the crucial roles of tryptophanyl-tRNA synthetase (TrpRS) and IDO1 on high-affinity Trp uptake. Finally, we discuss a possible molecular mechanism for TrpRS-mediated high-affinity Trp uptake into human cells.

## High-affinity Trp uptake causes immunosuppression

The high-affinity Trp transport system is up-regulated in human IFN-γ-treated and IDO1-expressing cells [5–8]. IDO1 mediates the initial rate-limiting step in the kynurenine (Kyn) pathway, which converts Trp to Kyn [9–11]. IDO1, which contains a heme prosthetic group, is predominantly found in the placenta and immune cells [12–14]. Expression of IDO1 is up-regulated by IFN-γ, which initiates a multi-signal response to neutralize pathogens and neoplasia [15–17]. Specific types of immune cells are suppressed by Trp depletion itself as well as compounds related to the breakdown of Trp (e.g. Kyn) [12–14,18–28]. Indeed, it is well established that T cell proliferation is inhibited when Trp is depleted to concentrations <1 μM [13,29]. Thus, increased high-affinity Trp uptake into cells leads to extracellular Trp depletion, thereby eliciting an immunosuppressive action (Figure 1B) [12–14,18–28].

IDO1 performs numerous biological roles including controlling T cell propagation, regulating antitumor immunity, and maintaining maternal tolerance towards the allogeneic fetus [12–14,18–23]. For example, IDO1 expression is up-regulated in the placenta leading to Trp depletion at the maternal-fetal interface, which aids in protecting the fetus from rejection by the maternal immune system [12,27]. Human IDO is expressed in MDMs and specific monocyte-derived dendritic cells (DCs) [13,14]. The diminished extracellular levels of Trp resulting from IDO1-mediated Trp breakdown in MDMs and DCs blocks T cell proliferation, leading to immunosuppression [12–14,20,22,23]. The basal expression level of the high-affinity Trp-specific transport system is low in monocytes but undergoes selective induction upon the differentiation of MDMs [5]. This high-affinity transport system allows MDMs to efficiently take up Trp even at low substrate levels. A lack of IDO1 expression and/or activity has been shown to augment various autoimmune disorders [30]. Furthermore, IDO1 expression levels are significantly up-regulated in certain types of cancer and IFN-γ-stimulated cells [7,8,12–14,18–23,25,26]. Some cancers also take advantage of the Trp metabolism-mediated immunosuppressive effect to block host immune rejection [18,20,23–26,28], although other cancers are reported to require Trp for survival (i.e. similar to T cells) [31]. For example, it has been demonstrated that up-regulated levels of IDO1 in immunogenic mouse tumor cells block their rejection in preimmunized mice [18,20]. Furthermore, constitutive expression of IDO1 prevents tumor cells from being rejected by T cells [18,20]. Intriguingly, expression of the high-affinity Trp transport system in mouse and human tumor cells is induced by IDO1 [6]. As such, the high-affinity Trp uptake system could be a promising target for anticancer therapeutics.

Another example of an enzyme that breaks down Trp is tryptophan 2,3-dioxygenase (TDO2). TDO2, which is mainly expressed in the liver and contains a heme prosthetic group, converts Trp to Kyn. In addition, TDO2 is known to be expressed in cancer cells where it induces immune resistance [24,32].

## Both IDO1 and TrpRS, which are up-regulated by IFN-γ, are required for high-affinity Trp uptake into cells

Human TrpRS is unique amongst aminoacyl-tRNA synthetases in that its expression is up-regulated by IFN-γ [33–37]. Moreover, TrpRS expression is highly up-regulated during the maturation from human monocytes into MDMs or DCs [38,39]. Human IFN-γ stimulates expression of human TrpRS protein and its translocation into the nucleus or extracellular space [40–44]. Furthermore, analysis of the human transcriptome database shows marked overexpression of TrpRS in the placenta [45]. The expression of mouse TrpRS was also found to be up-regulated by mouse IFN-γ [46].

Additionally, high-affinity Trp uptake is suppressed by siRNA-mediated down-regulation of human TrpRS or IDO1 expression [8]. In contrast, overexpression of human TrpRS or IDO1 enhances high-affinity Trp uptake [8]. These results suggest that high-affinity Trp uptake relies on both IDO1 and TrpRS.

## Structure and function of human TrpRS

TrpRS is an aminoacyl-tRNA synthetase that mediates the ligation of Trp to its corresponding tRNA (tRNA^Trp^) in the cytosol [47,48]. This reaction entails the formation of tryptophanyl-AMP from Trp and ATP with the concurrent release of inorganic pyrophosphate (PPi) [47]. Transfer of the aminoacyl moiety of tryptophanyl-AMP to tRNA^Trp^ then yields tryptophanyl-tRNA^Trp^ and AMP [47]. The aminoacylation activity of mammalian TrpRS is regulated by its interaction with either Zn^2+^ or heme [49–53].

**Figure 2.**
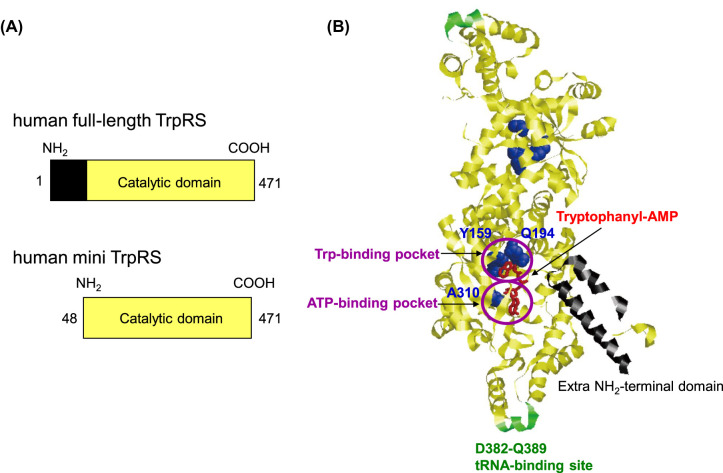
Structure of human TrpRS. (**A**) Schematic representation of human full-length and mini TrpRSs. The extra NH_2_-terminal domain of human TrpRS is shown in black. Numbers on the left and right correspond to the NH_2_- and COOH-terminal residues relative to human full-length TrpRS, respectively. (**B**) Tertiary structure of the dimeric human full-length TrpRS in complex with tryptophanyl-AMP (Protein Data Bank entry: 1R6T). The catalytic domain of human TrpRS is shown in yellow. Tryptophanyl-AMP is indicated in red. The extra NH_2_-terminal domain of human TrpRS is shown in black. Y159, Q194, and A310 residues are shown as space-filling models and colored in blue.

Two types of TrpRS are found in human cells. The predominant type is the full-length polypeptide (a.a. 1–471) along with a minor truncated version (mini TrpRS) (a.a. 48–471) where the first 47 residues from the NH_2_-terminal domain of TrpRS are missing as a result of alternative splicing (Figure 2A) [54,55]. IFN-γ markedly up-regulate the expression level of both types of TrpRSs in human cells [33–37]. Human mini TrpRS display almost the same aminoacylation activity as human full-length TrpRS [56].

TrpRS also exhibits functions that are distinct from aminoacylation, including cell-signaling cascades related to the immune system and angiogenesis [8,41,42,53,57–61]. Table 1 summarizes the functions of human full-length and mini TrpRSs. Both types of TrpRSs are secreted from cells [40,42,43,61,62]. Notably, however, mini TrpRS acts as an angiostatic factor although the full-length protein does not [57]. Specifically, secreted mini TrpRS binds to VE-cadherin thereby blocking VEGF-mediated interaction of VE-cadherin with VEGFR2 to inhibit angiogenesis [59,63–65]. A recent study reported that mini TrpRS also acts as an inhibitory ligand of neuropilin-1, which is essential for blood vessel development [62]. Moreover, full-length TrpRS mediates the activation of tumor suppressor p53 [41]. Full-length TrpRS translocates into the nucleus upon IFN-γ stimulation and promotes poly(ADP-ribosyl)ation of DNA-PKcs by interacting with the catalytic subunit of DNA-dependent protein kinase (DNA-PKcs) and poly(ADP-ribose) polymerase 1 (PARP-1) [41]. The activation of DNA-PKcs kinase brings about p53 phosphorylation thereby switching on its function [41]. In contrast, mini TrpRS cannot bind DNA-PKcs and PARP-1 [41].

**Table 1.** Examples of the function of human full-length and mini TrpRSs

Function	Full-length TrpRS	Mini TrpRS
tRNA aminoacylation activity	+	+
Secretion from cells	+	+
Angiostatic activity	−	+
Interaction with VE-cadherin	−	+
Interaction with PARP-1 and DNA-PKcs	+	−
Induction of high-affinity Trp uptake	+	+

## Human TrpRS mediates high-affinity Trp uptake

Akin to the high-affinity Trp uptake system, the expression of TrpRS is markedly increased in IFN-γ-treated cells and TrpRS has a strong selectivity and affinity for Trp [66,67]. TrpRS-specific siRNA has been shown to inhibit the expression of human TrpRS, which then decreases high-affinity Trp uptake [8]. In contrast, high-affinity Trp uptake is enhanced by up-regulated expression of human TrpRS [8]. Moreover, the addition of purified TrpRS into assay buffer including living cells increased the rate of high-affinity Trp uptake [8]. Both the full-length and mini form of human TrpRS stimulate high-affinity Trp uptake (Table 1) [8]. These observations demonstrate that human TrpRS plays a central role in regulating high-affinity Trp uptake into human cells.

## Trp depletion, induced by Trp-metabolizing enzymes, enhances TrpRS-mediated high-affinity Trp uptake

Cellular expression of IDO1 leads to Trp starvation, resulting in increased amounts of activating transcription factor 4 (ATF4) protein [68–70]. ATF4 is a stress-induced transcription factor [71]. Its expression is known to be enhanced by amino acid starvation [72–74]. Up-regulation of IDO1 significantly increases high-affinity Trp uptake upon addition of purified TrpRS protein into the assay buffer [70]. In contrast, overexpression of a mutant form of IDO1 (H346A), which cannot bind heme or convert Trp to Kyn [10], has no discernable influence on TrpRS-mediated Trp uptake into cells [70]. Furthermore, overexpression of TDO2 also stimulates ATF4 expression, which markedly increases extracellular TrpRS-mediated high-affinity Trp uptake [70]. Despite catalyzing the same reaction, IDO1 and TDO2 display discrete enzymatic properties [17,75–77]. Amino acid sequence similarity between IDO1 and TDO2 is low and although IDO1 is a monomer TDO2 exists as a homotetrameric form. Moreover, the overall tertiary architecture and relative 3D orientation of the small domains are dissimilar in human IDO1 and TDO2 [11,78,79]. The evident disparity between IDO1 and TDO2 suggests that high-affinity Trp uptake does not rely on protein-protein interactions involving these two enzymes.

Addition of TrpRS protein to Trp-starved cells incubated in Trp-free medium results in a marked enhancement in high-affinity Trp uptake, akin to cells expressing IDO1or TDO2 [70]. In contrast, Kyn has no effect on high-affinity Trp uptake brought about by extracellular TrpRS [70]. Moreover, high-affinity Trp uptake into human TrpRS-overexpressing cells is also significantly enhanced upon Trp starvation [80]. In conclusion, Trp starvation is crucial for high-affinity Trp uptake not only into cells to which TrpRS protein has been added but also into cells that are overexpressing TrpRS.

## TrpRS increases high-affinity Trp uptake via the biosynthesis of tryptophanyl-AMP

Tertiary structural position of amino acid residues crucial for Trp-, ATP- and tRNA-binding in human full-length TrpRS is shown in Figure 2B. Overexpression of a double mutant of TrpRS (Y159A/Q194A), which does not bind Trp, or a single mutant of TrpRS (A310W), in which the ATP-binding pocket is blocked [58], did not enhance Trp uptake into either normal or Trp-starved cells [8,80]. Importantly, both these TrpRS mutants cannot generate tryptophanyl-AMP. A truncated mutant of TrpRS (Δ382–389), in which amino acid residues 382–389 are missing, is capable of generating tryptophanyl-AMP but unable to aminoacylate tRNA because it cannot bind tRNA [81]. Overexpression of TrpRS (Δ382–389) increases Trp uptake into both normal and Trp-starved cells at levels equivalent to that observed in cells overexpressing wild-type TrpRS [8,80]. These findings indicate that unlike the ability of human TrpRS to bind tRNA, its ability to bind Trp and ATP are an absolute requirement for high-affinity Trp uptake into Trp-starved cells.

Trp uptake into human cells overexpressing TrpRS that were starved of Trp was significantly inhibited by the addition of PPi [80]. The presence of free PPi can disturb the equilibrium of the initial reaction in favor of breaking down aminoacyl-AMP, thereby blocking transfer of the amino acid to the tRNA. The presence of extracellular human TrpRS enhances Trp uptake into Trp-starved cells, but this uptake is inhibited by addition of PPi into the assay buffer [80]. These findings suggest that TrpRS orchestrates high-affinity Trp uptake via the generation of tryptophanyl-AMP.

## Proposed molecular mechanism for high-affinity Trp uptake induced by IFN-γ

**Figure 3.**
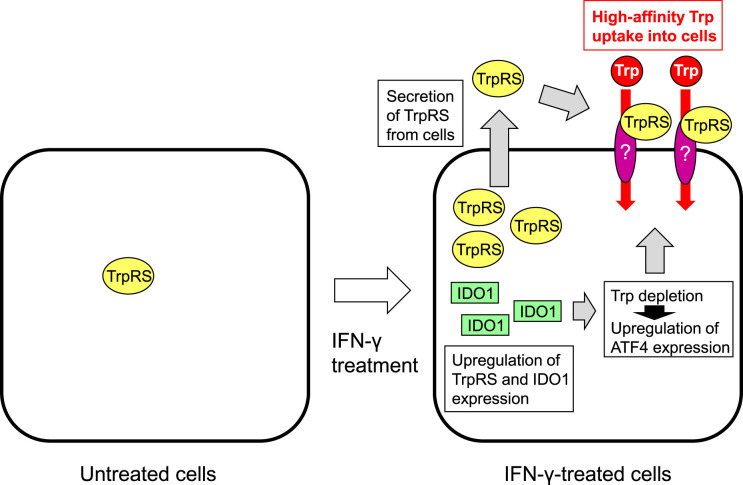
A schematic model of the regulatory mechanism of TrpRS-mediated high-affinity Trp uptake upon IFN-γ treatment of human cells. IFN-γ stimulates expression of both IDO1 and TrpRS. Up-regulated levels of IDO1 leads to Trp depletion, which in turn induces up-regulation of ATF4 expression. Secreted TrpRS will bind to Trp in the extracellular environment and regulate Trp uptake into human cells.

The proposed model for TrpRS-mediated high-affinity Trp uptake into human cells elicited by IFN-γ is depicted in Figure 3. IFN-γ brings about increased expression of both IDO1 and TrpRS [8,15,17,33–37]. Up-regulated levels of IDO1 enhances the metabolism of Trp, leading to Trp deficiency, which in turn induces up-regulation of ATF4. Additional work is required to identify molecules at the cell surface that interact with extracellular TrpRS and determine whether these molecules are produced as a result of intracellular Trp depletion. Moreover, it has been reported that some TrpRS is secreted [82] and that extracellular TrpRS is present in the cell culture medium [40,42,43]. A recent study reported that human TrpRS is secreted from cells after infection with a pathogen as part of a defense mechanism [42]. Additional investigations are required to investigate whether cellular secretion of TrpRS is increased upon Trp starvation. It is proposed that secreted TrpRS will bind to Trp in the extracellular environment, which may lead to selective transportation of Trp into the cell via the TrpRS-interacting cell-surface molecules. Further studies are necessary to establish whether the novel high-affinity Trp transport system comprises TrpRS itself or an unidentified transporter modulated by TrpRS.

## Future directions

The biosynthesis of tryptophanyl-AMP has been shown to be crucial for TrpRS-mediated high-affinity Trp uptake [80]. Nonetheless, the precise mechanism by which tryptophanyl-AMP mediates Trp uptake remains to be established. It was recently reported that aminoacyl-tRNA synthetases produce aminoacyl-AMP and act as aminoacyl transferases to modify the ɛ-amino group of L-lysine (Lys) residues in proteins [83–85]. This posttranslational modification can subsequently be eliminated by NAD^+^-dependent protein deacetylases (e.g. sirtuins) [83]. Given that the biosynthesis of tryptophanyl-AMP is required for high-affinity Trp uptake [80], TrpRS could facilitate this Trp uptake by protein tryptophanylation. For instance, extracellular TrpRS could tryptophanylate specific cell surface proteins that then initiate high-affinity Trp uptake. Additional studies are needed to determine whether protein tryptophanylation mediated by TrpRS is involved in cellular Trp uptake.

Our previous work demonstrated that Kyn, a physiological agonist of the aryl hydrocarbon receptor (AhR) [86], did not play a major role in high-affinity Trp uptake facilitated by extracellular TrpRS [70]. Nonetheless, some cell types are reported to display enhanced high-affinity Trp uptake when treated with Kyn [7]. A recent study shows low levels of Trp sensitizes the AhR pathway by up-regulating AhR expression [87]. Thus, additional investigations are required to establish whether the AhR pathway is implicated in the molecular mechanism of high-affinity Trp uptake using various cell types.

Starvation of most amino acids is reported to induce ATF4 expression and enhance inactivation of a mechanistic target of rapamycin complex 1 (mTORC1), leading to induction of autophagy [88,89]. Trp depletion also stimulates ATF4 expression [68–70]. However, high-affinity Trp uptake, which is enhanced under Trp-starved conditions, into TrpRS-overexpressing cells is markedly reduced by incubation of the cells with Torin-1 [80], a powerful inhibitor of mTORC1/2 and an effective inducer of autophagy [90]. These findings imply that Trp depletion does not enhance the inactivation of mTORC1/2. This mechanism is unique to Trp. It was recently demonstrated that ribosomes in Trp-depleted cells bypass Trp codons of mRNA and Trp-to-Phe codon reassignment occurs [90–92]. Intringuingly, Torin-1 was found to suppress ribosomal frameshifting in Trp-starved cells [90]. Thus, suppression of frameshifting and Trp-to-Phe codon reassignment by Torin-1 may have decreased high-affinity Trp uptake into Trp-starved cells. Given that these changes are specific to conditions of Trp starvation, they may be associated with high-affinity Trp uptake.

## Concluding remarks

Both TrpRS and IDO1, which are up-regulated by IFN-γ, are crucial for high-affinity Trp uptake into cells. Overexpression of IDO1 or TDO2 increases this TrpRS-mediated Trp uptake. Moreover, a deficiency in Trp induced by IDO1 or TDO2 is critical for TrpRS-mediated high-affinity Trp uptake into cells. It should be emphasized that TrpRS facilitates high-affinity uptake via the generation of tryptophanyl-AMP. Additional data is needed to reveal the precise molecular mechanism of high-affinity Trp uptake via extracellular TrpRS, particularly under Trp-depleted conditions. Trp uptake is also utilized by certain types of cancer to prevent immune rejection by T cells. Thus, proteins related to high-affinity Trp uptake represent potential targets for new anticancer agents.

## Perspectives

- High-affinity Trp uptake into human cells leads to extracellular Trp depletion, thereby eliciting an immunosuppressive action.
- Both IDO1 and TrpRS, which are up-regulated by IFN-γ, play a central role in high-affinity Trp uptake. Trp deficiency induced by IDO1 is critical for the Trp uptake and TrpRS facilitates the Trp uptake via the biosynthesis of tryptophanyl-AMP.
- Proteins related to high-affinity Trp uptake could be a promising target for anticancer agents.

## Open Access

Open access for this article was enabled by the participation of University of Tokyo in an all-inclusive *Read & Publish* agreement with Portland Press and the Biochemical Society under a transformative agreement with Individual.

## Abbreviations

AhR: aryl hydrocarbon receptor;
AMP: adenosine monophosphate
ATF4: activating transcription factor 4
ATP: adenosine triphosphate
BCH: 2-aminobicyclo-(2,2,1)-heptane-2-carboxylic acid
DC: dendritic cell
DNA-PKcs: catalytic subunit of DNA-dependent protein kinase
His: L-histidine
IDO1: indoleamine 2,3-dioxygenase 1
IFN: interferon
Ile: L-isoleucine
Kyn: kynurenine
LAT: L-type amino acid transporter
Leu: L-leucine
Lys: L-lysine
MDM: monocyte-derived macrophage
Met: L-methionine
mRNA: messenger RNA
mTORC1: mechanistic target of rapamycin complex 1
PARP1: poly(ADP-ribose) polymerase 1
Phe: L-phenylalanine
PPi: pyrophosphate
TDO2: tryptophan 2,3-dioxygenase
tRNA: transfer RNA
Trp: L-tryptophan
TrpRS: tryptophanyl-tRNA synthetase
Tyr: L-tyrosine
Val: L-valine

## References

[1] Segawa, H., Fukasawa, Y., Miyamoto, K., Takeda, E., Endou, H. and Kanai, Y. (1999) Identification and functional characterization of a Na^+^-independent neutral amino acid transporter with broad substrate selectivity. J. Biol. Chem. 274, 19745–19751 10.1074/jbc.274.28.19745 10391916

[2] Yanagida, O., Kanai, Y., Chairoungdua, A., Kim, D.K., Segawa, H., Nii, T. et al. (2001) Human L-type amino acid transporter 1 (LAT1): characterization of function and expression in tumor cell lines. Biochim. Biophys. Acta 1514, 291–302 10.1016/s0005-2736(01)00384-4 11557028

[3] del Amo, E.M., Urtti, A. and Yliperttula, M. (2008) Pharmacokinetic role of L-type amino acid transporters LAT1 and LAT2. Eur. J. Pharm. Sci. 35, 161–174 10.1016/j.ejps.2008.06.015 18656534

[4] Kudo, Y., Boyd, C.A.R., Sargent, I.L. and Redman, C.W.G. (2003) Decreased tryptophan catabolism by placental indoleamine 2,3-dioxygenase in preeclampsia. Am. J. Obstet. Gynecol. 188, 719–726 10.1067/mob.2003.156 12634647

[5] Seymour, R.L., Ganapathy, V., Mellor, A.L. and Munn, D.H. (2006) A high-affinity, tryptophan-selective amino acid transport system in human macrophages. J. Leukoc. Biol. 80, 1320–1327 10.1189/jlb.1205727 16997853

[6] Silk, J.D., Lakhal, S., Laynes, R., Vallius, L., Karydis, I., Marcea, C. et al. (2011) IDO induces expression of a novel tryptophan transporter in mouse and human tumor cells. J. Immunol. 187, 1617–1625 10.4049/jimmunol.1000815 21742973 PMC3512081

[7] Bhutia, Y.D., Babu, E. and Ganapathy, V. (2015) Interferon-γ induces a tryptophan-selective amino acid transporter in human colonic epithelial cells and mouse dendritic cells. Biochim. Biophys. Acta 1848, 453–462 10.1016/j.bbamem.2014.10.021 25450809

[8] Miyanokoshi, M., Yokosawa, T. and Wakasugi, K. (2018) Tryptophanyl-tRNA synthetase mediates high-affinity tryptophan uptake into human cells. J. Biol. Chem. 293, 8428–8438 10.1074/jbc.ra117.001247 29666190 PMC5986205

[9] Shimizu, T., Nomiyama, S., Hirata, F. and Hayaishi, O. (1978) Indoleamine 2,3-dioxygenase. Purification and some properties. J. Biol. Chem. 253, 4700–4706 10.1016/S0021-9258(17)30447-7 26687

[10] Littlejohn, T.K., Takikawa, O., Truscott, R.J. and Walker, M.J. (2003) Asp^274^ and His^346^ are essential for heme binding and catalytic function of human indoleamine 2,3-dioxygenase. J. Biol. Chem. 278, 29525–29531 10.1074/jbc.m301700200 12766158

[11] Sugimoto, H., Oda, S., Otsuki, T., Hino, T., Yoshida, T. and Shiro, Y. (2006) Crystal structure of human indoleamine 2,3-dioxygenase: catalytic mechanism of O_2_ incorporation by a heme-containing dioxygenase. Proc. Natl Acad. Sci. U.S.A. 103, 2611–2616 10.1073/pnas.0508996103 16477023 PMC1413787

[12] Munn, D.H., Zhou, M., Attwood, J.T., Bondarev, I., Conway, S.J., Marshall, B. et al. (1998) Prevention of allogeneic fetal rejection by tryptophan catabolism. Science 281, 1191–1193 10.1126/science.281.5380.1191 9712583

[13] Munn, D.H., Shafizadeh, E., Attwood, J.T., Bondarev, I., Pashine, A. and Mellor, A.L. (1999) Inhibition of T cell proliferation by macrophage tryptophan catabolism. J. Exp. Med. 189, 1363–1372 10.1084/jem.189.9.1363 10224276 PMC2193062

[14] Terness, P., Bauer, T.M., Röse, L., Dufter, C., Watzlik, A., Simon, H. et al. (2002) Inhibition of allogeneic T cell proliferation by indoleamine 2,3-dioxygenase-expressing dendritic cells: mediation of suppression by tryptophan metabolites. J. Exp. Med. 196, 447–457 10.1084/jem.20020052 12186837 PMC2196057

[15] Yoshida, R., Imanishi, J., Oku, T., Kishida, T. and Hayaishi, O. (1981) Induction of pulmonary indoleamine 2,3-dioxygenase by interferon. Proc. Natl Acad. Sci. U.S.A. 78, 129–132 10.1073/pnas.78.1.129 6165986 PMC319004

[16] Nathan, C.F., Murray, H.W., Wiebe, M.E. and Rubin, B.Y. (1983) Identification of interferon-γ as the lymphokine that activates human macrophage oxidative metabolism and antimicrobial activity. J. Exp. Med. 158, 670–689 10.1084/jem.158.3.670 6411853 PMC2187114

[17] Taylor, M.W. and Feng, G. (1991) Relationship between interferon-γ, indoleamine 2,3-dioxygenase, and tryptophan catabolism. FASEB J. 5, 2516–2522 10.1096/fasebj.5.11.1907934 1907934

[18] Friberg, M., Jennings, R., Alsarraj, M., Dessureault, S., Cantor, A., Extermann, M. et al. (2002) Indoleamine 2,3-dioxygenase contributes to tumor cell evasion of T cell-mediated rejection. Int. J. Cancer 101, 151–155 10.1002/ijc.10645 12209992

[19] Munn, D.H., Sharma, M.D., Lee, J.R., Jhaver, K.G., Johnson, T.S., Keskin, D.B. et al. (2002) Potential regulatory function of human dendritic cells expressing indoleamine 2,3-dioxygenase. Science 297, 1867–1870 10.1126/science.1073514 12228717

[20] Uyttenhove, C., Pilotte, L., Théate, I., Stroobant, V., Colau, D., Parmentier, N. et al. (2003) Evidence for a tumoral immune resistance mechanism based on tryptophan degradation by indoleamine 2,3-dioxygenase. Nat. Med. 9, 1269–1274 10.1038/nm934 14502282

[21] Meisel, R., Zibert, A., Laryea, M., Göbel, U., Däubener, W. and Dilloo, D. (2004) Human bone marrow stromal cells inhibit allogeneic T-cell responses by indoleamine 2,3-dioxygenase-mediated tryptophan degradation. Blood 103, 4619–4621 10.1182/blood-2003-11-3909 15001472

[22] Mellor, A.L. and Munn, D.H. (2004) IDO expression by dendritic cells: tolerance and tryptophan catabolism. Nat. Rev. Immunol. 4, 762–774 10.1038/nri1457 15459668

[23] Munn, D.H. and Mellor, A.L. (2004) IDO and tolerance to tumors. Trends Mol. Med. 10, 15–18 10.1016/j.molmed.2003.11.003 14720581

[24] Pilotte, L., Larrieu, P., Stroobant, V., Colau, D., Dolusic, E., Frédérick, R. et al. (2012) Reversal of tumoral immune resistance by inhibition of tryptophan 2,3-dioxygenase. Proc. Natl Acad. Sci. U.S.A. 109, 2497–2502 10.1073/pnas.1113873109 22308364 PMC3289319

[25] van Baren, N. and Van den Eynde, B.J. (2015) Tryptophan-degrading enzymes in tumoral immune resistance. Front. Immunol. 6, 34 10.3389/fimmu.2015.00034 25691885 PMC4315104

[26] Munn, D.H. and Mellor, A.L. (2016) IDO in the tumor microenvironment: inflammation, counter-regulation, and tolerance. Trends Immunol. 37, 193–207 10.1016/j.it.2016.01.002 26839260 PMC4916957

[27] Wei, H., Liu, S., Lian, R., Huang, C., Li, Y., Chen, L. et al. (2020) Abnormal expression of indoleamine 2, 3-dioxygenase in human recurrent miscarriage. Reprod. Sci. 27, 1656–1664 10.1007/s43032-020-00196-5 32430712

[28] Zhao, Y., Tao, F., Jiang, J., Chen, L., Du, J., Cheng, X. et al. (2021) Tryptophan 2, 3-dioxygenase promotes proliferation, migration and invasion of ovarian cancer cells. Mol. Med. Rep. 23, 445 10.3892/mmr.2021.12084 33846800 PMC8060793

[29] Kudo, Y., Boyd, C.A.R., Sargent, I.L. and Redman, C.W.G. (2001) Tryptophan degradation by human placental indoleamine 2,3-dioxygenase regulates lymphocyte proliferation. J. Physiol. 535, 207–215 10.1111/j.1469-7793.2001.00207.x 11507170 PMC2288791

[30] Kwidzinski, E., Bunse, J., Aktas, O., Richter, D., Mutlu, L., Zipp, F. et al. (2005) Indolamine 2,3-dioxygenase is expressed in the CNS and down-regulates autoimmune inflammation. FASEB J. 19, 1347–1349 10.1096/fj.04-3228fje 15939737

[31] Kenski, J.C.N., Huang, X., Vredevoogd, D.W., de Bruijn, B., Traets, J.J.H., Ibáñez-Molero, S. et al. (2023) An adverse tumor-protective effect of IDO1 inhibition. Cell Rep. Med. 4, 100941 10.1016/j.xcrm.2023.100941 36812891 PMC9975322

[32] Schmidt, S.K., Müller, A., Heseler, K., Woite, C., Spekker, K., MacKenzie, C.R. et al. (2009) Antimicrobial and immunoregulatory properties of human tryptophan 2,3-dioxygenase. Eur. J. Immunol. 39, 2755–2764 10.1002/eji.200939535 19637229

[33] Fleckner, J., Rasmussen, H.H. and Justesen, J. (1991) Human interferon γ potently induces the synthesis of a 55-kDa protein (γ2) highly homologous to rabbit peptide chain release factor and bovine tryptophanyl-tRNA synthetase. Proc. Natl Acad. Sci. U.S.A. 88, 11520–11524 10.1073/pnas.88.24.11520 1763065 PMC53167

[34] Kisselev, L., Frolova, L. and Haenni, A.L. (1993) Interferon inducibility of mammalian tryptophanyl-tRNA synthetase: new perspectives. Trends Biochem. Sci. 18, 263–267 10.1016/0968-0004(93)90178-p 7692626

[35] Fleckner, J., Martensen, P.M., Tolstrup, A.B., Kjeldgaard, N.O. and Justesen, J. (1995) Differential regulation of the human, interferon inducible tryptophanyl-tRNA synthetase by various cytokines in cell lines. Cytokine 7, 70–77 10.1006/cyto.1995.1009 7749068

[36] Shaw, A.C., Larsen, M.R., Roepstorff, P., Justesen, J., Christiansen, G. and Birkelund, S. (1999) Mapping and identification of interferon γ-regulated HeLa cell proteins separated by immobilized pH gradient two-dimensional gel electrophoresis. Electrophoresis 20, 984–993 10.1002/(SICI)1522-2683(19990101)20:4/5&lt;984::AID-ELPS984>3.0.CO;2-R 10344276

[37] Liu, J., Shue, E., Ewalt, K.L. and Schimmel, P. (2004) A new γ-interferon-inducible promoter and splice variants of an anti-angiogenic human tRNA synthetase. Nucleic Acids Res. 32, 719–727 10.1093/nar/gkh240 14757836 PMC373357

[38] Krause, S.W., Rehli, M., Kreutz, M., Schwarzfischer, L., Paulauskis, J.D. and Andreesen, R. (1996) Differential screening identifies genetic markers of monocyte to macrophage maturation. J. Leukoc. Biol. 60, 540–545 10.1002/jlb.60.4.540 8864140

[39] Matsunaga, T., Ishida, T., Takekawa, M., Nishimura, S., Adachi, M. and Imai, K. (2002) Analysis of gene expression during maturation of immature dendritic cells derived from peripheral blood monocytes. Scand. J. Immunol. 56, 593–601 10.1046/j.1365-3083.2002.01179.x 12472671

[40] Kapoor, M., Zhou, Q., Otero, F., Myers, C.A., Bates, A., Belani, R. et al. (2008) Evidence for annexin II-S100A10 complex and plasmin in mobilization of cytokine activity of human TrpRS. J. Biol. Chem. 283, 2070–2077 10.1074/jbc.m706028200 17999956

[41] Sajish, M., Zhou, Q., Kishi, S., Valdez, Jr, D.M., Kapoor, M., Guo, M. et al. (2012) Trp-tRNA synthetase bridges DNA-PKcs to PARP-1 to link IFN-γ and p53 signaling. Nat. Chem. Biol. 8, 547–554 10.1038/nchembio.937 22504299 PMC3780985

[42] Ahn, Y.H., Park, S., Choi, J.J., Park, B.K., Rhee, K.H., Kang, E. et al. (2016) Secreted tryptophanyl-tRNA synthetase as a primary defence system against infection. Nat. Microbiol. 2, 16191 10.1038/nmicrobiol.2016.191 27748732

[43] Nguyen, T.T.T., Choi, Y.H., Lee, W.-K., Ji, Y., Chun, E., Kim, Y.H. et al. (2023) Tryptophan-dependent and -independent secretions of tryptophanyl- tRNA synthetase mediate innate inflammatory responses. Cell Rep. 42, 111905 10.1016/j.celrep.2022.111905 36640342

[44] Lazar, I., Livneh, I., Ciechanover, A. and Fabre, B. (2024) Tryptophanyl-transfer RNA synthetase is involved in a negative feedback loop mitigating interferon-γ-induced gene expression. Cells 13, 180 10.3390/cells13020180 38247871 PMC10813977

[45] Ewalt, K.L. and Schimmel, P. (2002) Activation of angiogenic signaling pathways by two human tRNA synthetases. Biochemistry 41, 13344–13349 10.1021/bi020537k 12416978

[46] Miyanokoshi, M., Tanaka, T., Tamai, M., Tagawa, Y. and Wakasugi, K. (2013) Expression of the rodent-specific alternative splice variant of tryptophanyl-tRNA synthetase in murine tissues and cells. Sci. Rep. 3, 3477 10.1038/srep03477 24327169 PMC3858792

[47] Schimmel, P.R. and Söll, D. (1979) Aminoacyl-tRNA synthetases: general features and recognition of transfer RNAs. Annu. Rev. Biochem. 48, 601–648 10.1146/annurev.bi.48.070179.003125 382994

[48] Schimmel, P. (1987) Aminoacyl-tRNA synthetases: general scheme of structure-functional relationships in the polypeptides and recognition of transfer RNAs. Annu. Rev. Biochem. 56, 125–158 10.1146/annurev.bi.56.070187.001013 3304131

[49] Kisselev, L.L., Favorova, O.O., Nurbekov, M.K., Dmitriyenko, S.G. and Engelhardt, W.A. (1981) Bovine tryptophanyl-tRNA synthetase. A zinc metalloenzyme. Eur. J. Biochem. 120, 511–517 10.1111/j.1432-1033.1981.tb05729.x 7333276

[50] Wakasugi, K. (2007) Human tryptophanyl-tRNA synthetase binds with heme to enhance its aminoacylation activity. Biochemistry 46, 11291–11298 10.1021/bi7012068 17877375

[51] Wakasugi, K. (2010) Species-specific differences in the regulation of the aminoacylation activity of mammalian tryptophanyl-tRNA synthetases. FEBS Lett. 584, 229–232 10.1016/j.febslet.2009.11.073 19941862

[52] Xu, X., Zhou, H., Zhou, Q., Hong, F., Vo, M.N., Niu, W. et al. (2018) An alternative conformation of human TrpRS suggests a role of zinc in activating non-enzymatic function. RNA Biol. 15, 649–658 10.1080/15476286.2017.1377868 28910573 PMC6103731

[53] Wakasugi, K. and Yokosawa, T. (2020) Non-canonical functions of human cytoplasmic tyrosyl-, tryptophanyl- and other aminoacyl-tRNA synthetases. Enzymes 48, 207–242 10.1016/bs.enz.2020.04.001 33837705

[54] Tolstrup, A.B., Bejder, A., Fleckner, J. and Justesen, J. (1995) Transcriptional regulation of the interferon-γ-inducible tryptophanyl-tRNA synthetase includes alternative splicing. J. Biol. Chem. 270, 397–403 10.1074/jbc.270.1.397 7814400

[55] Turpaev, K.T., Zakhariev, V.M., Sokolova, I.V., Narovlyansky, A.N., Amchenkova, A.M., Justesen, J. et al. (1996) Alternative processing of the tryptophanyl-tRNA synthetase mRNA from interferon treated human cells. Eur. J. Biochem. 240, 732–737 10.1111/j.1432-1033.1996.0732h.x 8856077

[56] Wakasugi, K., Nakano, T. and Morishima, I. (2005) Oxidative stress-responsive intracellular regulation specific for the angiostatic form of human tryptophanyl-tRNA synthetase. Biochemistry 44, 225–232 10.1021/bi048313k 15628863

[57] Wakasugi, K., Slike, B.M., Hood, J., Otani, A., Ewalt, K.L., Friedlander, M. et al. (2002) A human aminoacyl-tRNA synthetase as a regulator of angiogenesis. Proc. Natl Acad. Sci. U.S.A. 99, 173–177 10.1073/pnas.012602099 11773626 PMC117534

[58] Zhou, Q., Kapoor, M., Guo, M., Belani, R., Xu, X., Kiosses, W.B. et al. (2010) Orthogonal use of a human tRNA synthetase active site to achieve multifunctionality. Nat. Struct. Mol. Biol. 17, 57–61 10.1038/nsmb.1706 20010843 PMC3042952

[59] Nakamoto, T., Miyanokoshi, M., Tanaka, T. and Wakasugi, K. (2016) Identification of a residue crucial for the angiostatic activity of human mini tryptophanyl-tRNA synthetase by focusing on its molecular evolution. Sci. Rep. 6, 24750 10.1038/srep24750 27094087 PMC4837363

[60] Maras, J.S., Sharma, S., Bhat, A., Rooge, S., Aggrawal, R., Gupta, E. et al. (2021) Multi-omics analysis of respiratory specimen characterizes baseline molecular determinants associated with SARS-CoV-2 outcome. iScience 24, 102823 10.1016/j.isci.2021.102823 34308298 PMC8268673

[61] Kim, Y.T., Huh, J.W., Choi, Y.H., Yoon, H.K., Nguyen, T.T., Chun, E. et al. (2024) Highly secreted tryptophanyl tRNA synthetase 1 as a potential theranostic target for hypercytokinemic severe sepsis. EMBO Mol. Med. 16, 40–63 10.1038/s44321-023-00004-y 38177528 PMC10883277

[62] Gioelli, N., Neilson, L., Wei, N., Villari, G., Chen, W., Kuhle, B. et al. (2022) Neuropilin 1 and its inhibitory ligand mini-tryptophanyl-tRNA synthetase inversely regulate VE-cadherin turnover and vascular permeability. Nat. Commun. 13, 4188 10.1038/s41467-022-31904-1 35858913 PMC9300702

[63] Tzima, E., Reader, J.S., Irani-Tehrani, M., Ewalt, K.L., Schwartz, M.A. and Schimmel, P. (2005) VE-cadherin links tRNA synthetase cytokine to anti-angiogenic function. J. Biol. Chem. 280, 2405–2408 10.1074/jbc.c400431200 15579907

[64] Tzima, E. and Schimmel, P. (2006) Inhibition of tumor angiogenesis by a natural fragment of a tRNA synthetase. Trends Biochem. Sci. 31, 7–10 10.1016/j.tibs.2005.11.002 16297628

[65] Zeng, R., Chen, Y.C., Zeng, Z., Liu, X.X., Liu, R., Qiang, O. et al. (2012) Inhibition of mini-TyrRS-induced angiogenesis response in endothelial cells by VE-cadherin-dependent mini-TrpRS. Heart Vessels 27, 193–201 10.1007/s00380-011-0137-1 21442253

[66] Kisselev, L.L., Favorova, O.O. and Kovaleva, G.K. (1979) Tryptophanyl-tRNA synthetase from beef pancreas. Methods Enzymol. 59, 234–257 10.1016/0076-6879(79)59087-9 86935

[67] Ewalt, K.L., Yang, X.L., Otero, F.J., Liu, J., Slike, B. and Schimmel, P. (2005) Variant of human enzyme sequesters reactive intermediate. Biochemistry 44, 4216–4221 10.1021/bi048116l 15766249

[68] Timosenko, E., Ghadbane, H., Silk, J.D., Shepherd, D., Gileadi, U., Howson, L.J. et al. (2016) Nutritional stress induced by tryptophan-degrading enzymes results in ATF4-dependent reprogramming of the amino acid transporter profile in tumor cells. Cancer Res. 76, 6193–6204 10.1158/0008-5472.can-15-3502 27651314 PMC5096689

[69] Adam, I., Dewi, D.L., Mooiweer, J., Sadik, A., Mohapatra, S.R., Berdel, B. et al. (2018) Upregulation of tryptophanyl-tRNA synthethase adapts human cancer cells to nutritional stress caused by tryptophan degradation. Oncoimmunology 7, e1486353 10.1080/2162402x.2018.1486353 30524887 PMC6279332

[70] Yokosawa, T., Sato, A. and Wakasugi, K. (2020) Tryptophan depletion modulates tryptophanyl-tRNA synthetase-mediated high-affinity tryptophan uptake into human cells. Genes (Basel) 11, 1423 10.3390/genes11121423 33261077 PMC7760169

[71] Neill, G. and Masson, G.R. (2023) A stay of execution: ATF4 regulation and potential outcomes for the integrated stress response. Front. Mol. Neurosci. 16, 1112253 10.3389/fnmol.2023.1112253 36825279 PMC9941348

[72] Vattem, K.M. and Wek, R.C. (2004) Reinitiation involving upstream ORFs regulates ATF4 mRNA translation in mammalian cells. Proc. Natl Acad. Sci. U.S.A. 101, 11269–11274 10.1073/pnas.0400541101 15277680 PMC509193

[73] Kilberg, M.S., Shan, J. and Su, N. (2009) ATF4-dependent transcription mediates signaling of amino acid limitation. Trends Endocrinol. Metab. 20, 436–443 10.1016/j.tem.2009.05.008 19800252 PMC3587693

[74] Young, S.K. and Wek, R.C. (2016) Upstream open reading frames differentially regulate gene-specific translation in the integrated stress response. J. Biol. Chem. 291, 16927–16935 10.1074/jbc.r116.733899 27358398 PMC5016099

[75] Batabyal, D. and Yeh, S.R. (2007) Human tryptophan dioxygenase: a comparison to indoleamine 2,3-dioxygenase. J. Am. Chem. Soc. 129, 15690–15701 10.1021/ja076186k 18027945

[76] Thackray, S.J., Mowat, C.G. and Chapman, S.K. (2008) Exploring the mechanism of tryptophan 2,3-dioxygenase. Biochem. Soc. Trans. 36, 1120–1123 10.1042/bst0361120 19021508 PMC2652831

[77] Meng, B., Wu, D., Gu, J., Ouyang, S., Ding, W. and Liu, Z.J. (2014) Structural and functional analyses of human tryptophan 2,3-dioxygenase. Proteins 82, 3210–3216 10.1002/prot.24653 25066423

[78] Dick, R., Murray, B.P., Reid, M.J. and Correia, M.A. (2001) Structure-function relationships of rat hepatic tryptophan 2,3-dioxygenase: identification of the putative heme-ligating histidine residues. Arch. Biochem. Biophys. 392, 71–78 10.1006/abbi.2001.2420 11469796

[79] Lewis-Ballester, A., Forouhar, F., Kim, S.M., Lew, S., Wang, Y., Karkashon, S. et al. (2016) Molecular basis for catalysis and substrate-mediated cellular stabilization of human tryptophan 2,3-dioxygenase. Sci. Rep. 6, 35169 10.1038/srep35169 27762317 PMC5071832

[80] Yokosawa, T. and Wakasugi, K. (2023) Tryptophan-starved human cells overexpressing tryptophanyl-tRNA synthetase enhance high-affinity tryptophan uptake via enzymatic production of tryptophanyl-AMP. Int. J. Mol. Sci. 24, 15453 10.3390/ijms242015453 37895133 PMC10607379

[81] Yang, X.L., Otero, F.J., Ewalt, K.L., Liu, J., Swairjo, M.A., Köhrer, C. et al. (2006) Two conformations of a crystalline human tRNA synthetase-tRNA complex: implications for protein synthesis. EMBO J. 25, 2919–2929 10.1038/sj.emboj.7601154 16724112 PMC1500858

[82] Paley, E.L. (2011) Tryptamine-induced tryptophanyl-tRNA^trp^ deficiency in neurodifferentiation and neurodegeneration interplay: progenitor activation with neurite growth terminated in Alzheimer's disease neuronal vesicularization and fragmentation. J. Alzheimers Dis. 26, 263–298 10.3233/jad-2011-110176 21628792

[83] He, X.D., Gong, W., Zhang, J.N., Nie, J., Yao, C.F., Guo, F.S. et al. (2018) Sensing and transmitting intracellular amino acid signals through reversible lysine aminoacylations. Cell Metab. 27, 151–166 10.1016/j.cmet.2017.10.015 29198988

[84] Qin, R., Zhao, C., Wang, C.J., Xu, W., Zhao, J.Y., Lin, Y. et al. (2021) Tryptophan potentiates CD8^+^ T cells against cancer cells by TRIP12 tryptophanylation and surface PD-1 downregulation. J. Immunother. Cancer 9, e002840 10.1136/jitc-2021-002840 34326168 PMC8323461

[85] Sun, W.-X., Zhang, K.-H., Zhou, Q., Hu, S.-H., Lin, Y., Xu, W. et al. (2024) Tryptophanylation of insulin receptor by WARS attenuates insulin signaling. Cell. Mol. Life Sci. 81, 25 10.1007/s00018-023-05082-2 38212570 PMC11072365

[86] Opitz, C.A., Litzenburger, U.M., Sahm, F., Ott, M., Tritschler, I., Trump, S. et al. (2011) An endogenous tumour-promoting ligand of the human aryl hydrocarbon receptor. Nature 478, 197–203 10.1038/nature10491 21976023

[87] Solvay, M., Holfelder, P., Klaessens, S., Pilotte, L., Stroobant, V., Lamy, J. et al. (2023) Tryptophan depletion sensitizes the AHR pathway by increasing AHR expression and GCN2/LAT1-mediated kynurenine uptake, and potentiates induction of regulatory T lymphocytes. J. Immunother. Cancer 11, e006728 10.1136/jitc-2023-006728 37344101 PMC10314700

[88] Ge, M.-K., Zhang, C., Zhang, N., He, P., Cai, H.-Y., Li, S. et al. (2023) The tRNA-GCN2-FBXO22-axis-mediated mTOR ubiquitination senses amino acid insufficiency. Cell Metab. 35, 2216–2230 10.1016/j.cmet.2023.10.016 37979583

[89] Livneh, I., Cohen-Kaplan, V., Fabre, B., Abramovitch, I., Lulu, C., Nataraj, N.B. et al. (2023) Regulation of nucleo-cytosolic 26S proteasome translocation by aromatic amino acids via mTOR is essential for cell survival under stress. Mol. Cell 83, 3333–3346 10.1016/j.molcel.2023.08.016 37738964

[90] Champagne, J., Pataskar, A., Blommaert, N., Nagel, R., Wernaart, D., Ramalho, S. et al. (2021) Oncogene-dependent sloppiness in mRNA translation. Mol. Cell 81, 4709–4721 10.1016/j.molcel.2021.09.002 34562372

[91] Bartok, O., Pataskar, A., Nagel, R., Laos, M., Goldfarb, E., Hayoun, D. et al. (2021) Anti-tumour immunity induces aberrant peptide presentation in melanoma. Nature 590, 332–337 10.1038/s41586-020-03054-1 33328638

[92] Pataskar, A., Champagne, J., Nagel, R., Kenski, J., Laos, M., Michaux, J. et al. (2022) Tryptophan depletion results in tryptophan-to-phenylalanine substitutants. Nature 603, 721–727 10.1038/s41586-022-04499-2 35264796 PMC8942854

